# Leydig cell tumor in ovary of a German Shepherd bitch: An immunohistopathological study

**Published:** 2017-03-15

**Authors:** Ghasem Farjanikish, Ahmad Oryan

**Affiliations:** 1 *Department of Pathobiology, Faculty of Veterinary Medicine, Lorestan University, Khorram Abad, Iran; *; 2 *Department of Pathobiology, School of Veterinary Medicine, Shiraz University, Shiraz, Iran.*

**Keywords:** Dog, Immunohistopathology, Leydig cell tumor, Ovary

## Abstract

Leydig cell tumor as a sex-cord stromal tumor is a relatively uncommon ovarian tumor in bitch. A 10-year-old female German Shepherd dog was presented because of protrusion of a large tumor-like mass of 16 × 14 × 7 cm in dimensions from her vagina. After stabilization of the patient, the mass was removed surgically and concurrent ovariohysterectomy was also performed. Macroscopically, the healthy tissue of the right ovary was totally replaced by a homogeneous, brown and firm mass. The neoplasm was well-circumscribed and nodular and it was clearly demarcated from the healthy tissue. Histological examination revealed the presence of solid sheets and acinar structures composed of polyhedral to elongated cells. The neoplastic cells had large, eosinophilic, and vacuolated cytoplasms with round to oval nuclei and expressed vimentin on immunohistochemical examination. These gross, microscopic and immuno-histochemical features are characteristics of ovarian Leydig cell tumor.

## Introduction

Ovarian tumors are relatively uncommon in bitch. Their frequency ranges between 0.5 and 1.2% with an average of 1.0%, but their incidence is probably under-estimated.^1^ According to the world health organization, the ovarian tumors can histologically be classified as granulose theca cell stromal tumor, Sertoli-stromal cell tumor, sex cord tumor with annular tubules, gynandro-blastoma, unclassified and steroid cell tumors. The Sertoli-stromal cell tumor consists of Sertoli-Leydig cell tumors, as well as pure Sertoli cell or pure Leydig cell tumors. Leydig cell tumors account for 15.0% to 20.0% of the steroid cell tumors in human.^2^^,^^3^ The Leydig cell tumors of the ovary are composed of cells resembling those of the corpus luteum in all species. These tumors consist of multiple lobules of neoplastic cells separated by a well-vascularized connective tissue stroma. The neoplastic cells are polygonal with abundant granular eosinophilic cytoplasm containing lipid vacuoles.^4^ The present report describes the histopathological and immunohistochemical features of a Leydig cell tumor in a German Shepherd bitch.

## Case Description

**Fig. 1:**
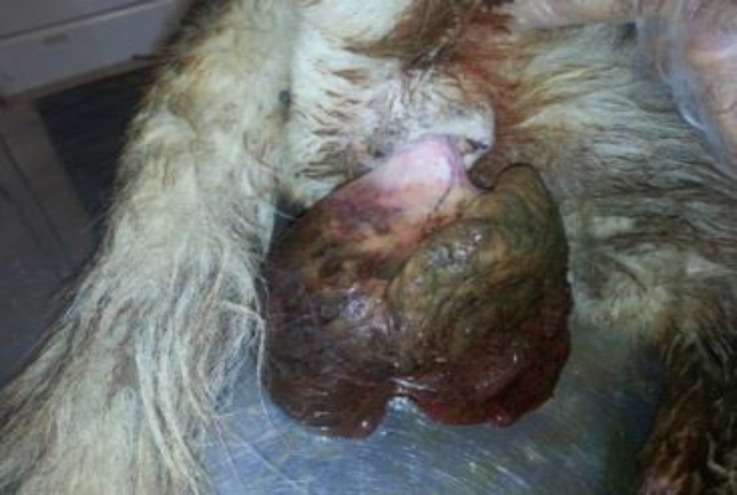
Leydig cell tumor in a 10-year-old bitch. A large mass measuring 16 × 14 × 7 cm protruded from the vagina

A 10-year-old female German Shepherd dog was admitted due to protrusion of a large mass of 16 × 14 × 7 cm in dimensions (Fig. 1) from her vagina which resulted in severe depression, lethargy and anorexia. During clinical examinations, pale mucous membrane, tachycardia, tachypnea and distention of the bladder, probably due to compression of the urethra by the mass were detected. After stabilization of the patient the mass was removed surgically and concurrent ovariohysterectomy was also performed. On gross examination, the healthy tissue of the right ovary was totally replaced by a tumor like mass. The mass was brown, homogeneous, firm and elastic at palpation. It was well-circumscribed, nodular and clearly demarcated from the healthy ovary.

The appropriate tissues were fixed in 10% neutral buffered formalin, dehydrated in graded ethanol, cleared in xylene and embedded in paraffin wax. Sections of 5 µm thicknesses were stained with hematoxylin and eosin (H & E) and studied by a compound light microscope. Serial sections were subjected to immunohistochemistry with primary monoclonal antibodies specific for pancyto-keratin, epithelial membrane antigen (EMA), S-100, inhibin, calretinin, synaptophysin, neuron-specific enolase (NSE), chromogranin and vimentin. All primary anti-bodies were from Novocastra Laboratories (Newcastle, UK). Labelling was detected with an avidin-biotin conjugate (ABC) procedure.

**Fig. 2:**
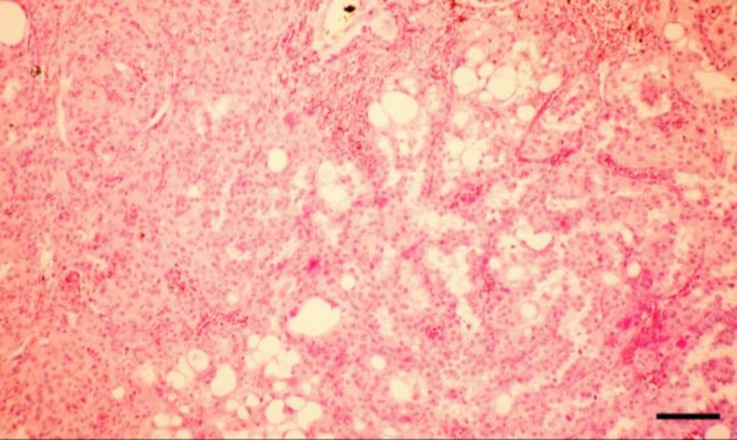
Microscopic examination reveals presence of solid sheets and acinar structures of Leydig cell tumor with polyhedral to elongated, vacuolated cells (H & E, Bar: 125 µm

**Fig. 3:**
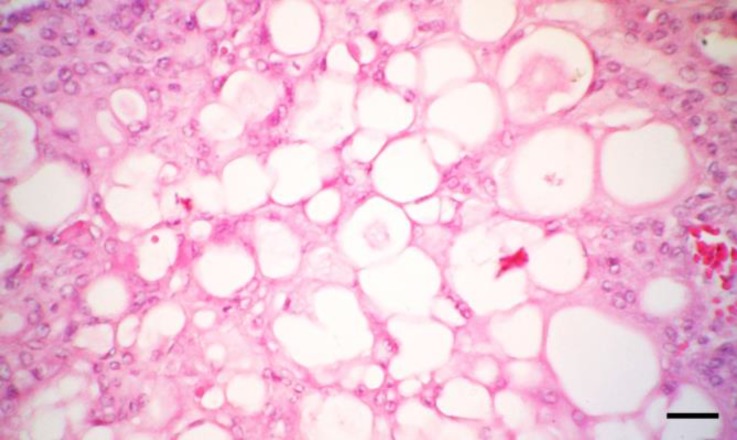
The neoplastic cells have eosinophilic and round to oval nuclei with single or multiple cytoplasmic vacuoles (H & E, Bar: 30 µm

Histological examination revealed presence of the solid sheets and acinar structures composed of polyhedral to elongated cells. Broad bands of fibrovascular stroma divided the encapsulated neoplastic mass into lobules of different sizes and shapes. The neoplastic cells had large, eosinophilic, and vacuolated cytoplasms and round to oval nuclei containing small nucleoli (Fig. 2). The single or multiple clear vacuoles were of different sizes and optically empty (Fig. 3). 

**Fig. 4:**
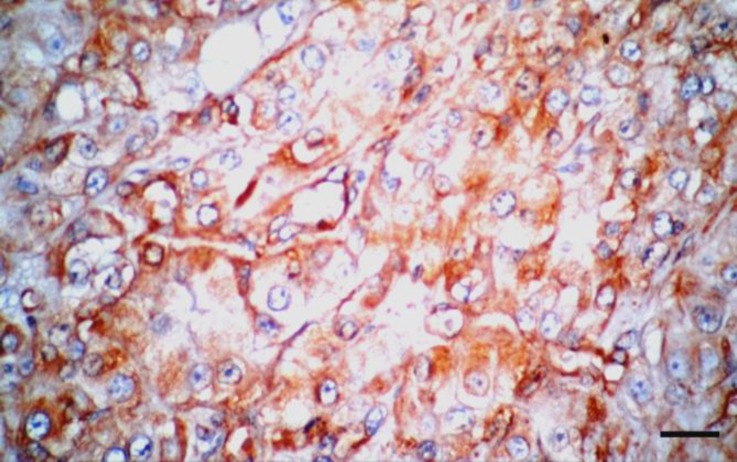
The tumor cells show cytoplasmic positivity for vimentin (IHC, Bar: 30 µm

In some parts of the tumor, angiogenesis was prominent which at some instances resulted in small hemorrhagic foci. The neoplastic cells labelled cytoplasmic positivity for vimentin (Fig. 4), but did not express any other marker. These immunohistochemical findings allowed confirming the diagnosis of Leydig cells tumor.

## Discussion

Canine ovarian neoplasms are uncommon.^5^^,^^6^ They occur more frequently in older, multiparous bitches or in bitches with ovarian remnant syndrome.^7^ Interstitial-cell tumors are relatively more common in male testes but the ovarian interstitial-cell tumors are rare in all species.^8^ Unlike the male dogs, the tumor is not common in ovaries of females. Leydig stromal tumor is usually benign, unilateral and metastasis of the tumor is very uncommon.^9^^,^^10^ During examination of the present case, pale mucous membrane, tachycardia, tachypnea and distension of the bladder probably due to pressure of the mass on urethra were detected. No details of the clinical signs have been provided in the ovarian interstitial-cell tumors that have previously been reported.^11^^,^^12^

An interstitial-cell tumor of the ovary resembles a corpus luteum both grossly and histologically.^13^ Micro-scopically, the cells typically have abundant eosinophilic cytoplasm; however, some cells may have vacuolated cytoplasm indicative of lipid. The nuclei are typically round with a single small nucleolus. The histologic description of the tumors in this case was similar to those described in cattle, eland, alpaca and dog.^13^^-^^15^

Most Leydig cell tumors show positive staining for vimentin.^15^^-^^18^ Other staining reactions that have been reported include staining for chromogranin, synapto-physin, S100 and cytokeratin.^16^ In the present report, the neoplastic cells labelled cytoplasmic positivity for vimentin. Ovariohysterectomy is a prudent method for preventing many diseases in dogs and cats, such as pyometra, mammary and ovarian tumors. In the present case, after stabilization of the patient, the mass was removed surgically and concurrent ovariohysterectomy was also performed.

Leydig cell tumor is a very rare neoplasma in the bitch. Veterinary practitioners should enlighten pet owners regarding the possibility of this neoplasm.^19^
